# Models of thermal motion in small-molecule crystallography

**DOI:** 10.1107/S2052252525004361

**Published:** 2025-06-06

**Authors:** Anna Hoser, Anders Ø. Madsen

**Affiliations:** a Faculty of Chemistry University of Warsaw Pasteura 1 Warsaw02-093 Poland; b Department of Pharmacy University of Copenhagen Copenhagen Denmark; Warsaw University, Poland

**Keywords:** Debye–Waller factor, thermal motion, anisotropic displacement parameters, lattice dynamics, normal-mode refinement, computational modelling, molecular crystals, dynamical simulations, charge, spin and momentum densities, pharmaceutical solids

## Abstract

The Debye–Waller factor, introduced a century ago, remains a fundamental component in the refinement of crystal structures against X-ray, neutron and electron diffraction data. This review marks its centenary by exploring its applications in small-molecule crystallography. We provide a historical overview of the development of the Debye–Waller factor and its foundations in lattice dynamics. The review discusses the practical use of anisotropic displacement parameters and their role in accurate structure determination. We also address the challenges and advancements in modelling thermal motion and disorder, the role of multi-temperature measurements and modern computational approaches.

## Abstract 2

This review commemorates the centenary of the Debye–Waller factor, highlighting its significance in quantifying the impact of thermal vibrations on scattering intensities as well as on crystal properties in small-molecule crystallography. It provides an introduction to thermal motion and displacement parameters, offering insights for chemists and crystallographers.

## 1. The Debye–Waller factor: 100 years of modelling

Soon after the famous discovery in 1912 that crystals diffract X-rays (Friedrich *et al.*, 1913 ▸), it became evident that the intensities of the diffracted X-rays depend on the temperature of the measurements (Bragg, 1914 ▸).

This variation was attributed to the thermal motion of atoms within the crystal lattice: the perfect crystal symmetry – where every atom can be related to equivalent atoms in other unit cells – is broken when the atoms wiggle about their mean positions: this causes a reduction in the diffracted intensities. The higher the temperature, the greater the reduction in the intensities observed.

At the same time as the discovery of X-ray diffraction, Max Born and Theodore von Kármán developed a theoretical model of atomic vibrations in crystals called ‘lattice dynamics’ (Born & von Kármán, 1912 ▸). In their approach, interacting atoms give rise to waves of motion (called phonons) throughout the crystal. This theoretical framework for understanding the vibrations of atoms in a crystal lattice provided the foundation for Peter Debye’s model of specific heat and phonon behaviour in solids (Debye, 1912 ▸), and led to the quantification of the effect of thermal vibrations on the scattering intensity (Debye, 1913 ▸) and this was furthered by Ivar Waller leading to the formulation of the Debye–Waller factor (Waller, 1923 ▸). The effect of temperature on the diffracted intensities was confirmed by more accurate measurements in the same period (James, 1925 ▸; James & Firth, 1927 ▸; Backhurst, 1922 ▸).

A century has passed, and the Debye–Waller factor (DW factor) still plays a central role in the models of crystals refined against X-ray, neutron and electron diffraction data.

In this contribution, we celebrate the centennial of the Debye–Waller factor by describing its applications in the context of small-molecule crystallography. In particular, we would like to give an introduction to thermal motion that is not too technical, and to provide pointers to places in the literature that we have found useful to further our understanding.

For many chemists that use crystallography as an analytical tool, the displacement parameters are mostly considered a convenient way to lower the refinement statistics and to help find accurate positions of the atoms. However, as we hope to convey in the following, there is a lot of information hidden in the analysis of Debye–Waller factors.

## 2. The Debye–Waller factor and anisotropic displacement parameters

Let us start by considering how the Debye–Waller factor is defined in the context of the usual scattering formalism used in X-ray crystallography. We consider the case of X-ray diffraction, but similar (though not identical) considerations apply to neutron and electron diffraction measurements. Atoms in a crystal lattice vibrate about their mean positions, even at the lowest temperatures because of zero-point motion. This implies that the measured diffraction intensities correspond to the scattering from an electron density 〈ρ(**r**)〉 in the unit cell that is time- and spatially-averaged over all unit cells in the crystal. The structure factor for reflection **h** becomes a Fourier transformation of this average electron density,
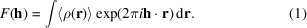
This average electron density can be approximated as a sum of individual atomic contributions. Each atom *m* can be described as the convolution of a static atomic density ρ_*m*_ and a function *p_m_* that describes the probability density of the position of the atom with respect to the atom’s reference position **r**_*m*0_. We sum up over all *N* atoms in the unit cell and multiply by each atom’s occupancy factor *n_m_*.
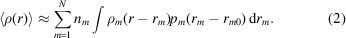
This approximation implies that the atomic electron densities ρ_*m*_ are smeared out with *p_m_* but are not deformed as a function of the displacements, a model that has been termed the ‘rigid pseudo atom model’.

The Fourier transform of the equation above gives the conventional approximation for the structure factor of a Bragg reflection:
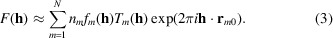
The scattering for each atom is a product of the atomic form factor *f_m_*(**h**), representing the scattering from a static picture (static electron density in the case of X-rays, static nucleus in the case of neutrons and a static electrostatic potential in the case of electron scattering). The Debye–Waller factor *T_m_*(**h**) is the Fourier transform of the probability density function
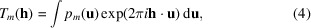
where **u** = **r**_*m*_ − **r**_*m*_0. *T_m_*(**h**) accounts for the reduction in the amplitude of the structure factor due to the averaging of this static object in both time and space between different unit cells. This smearing can be due to thermal motion but also due to static or dynamic disorder.

**Figure 1:**
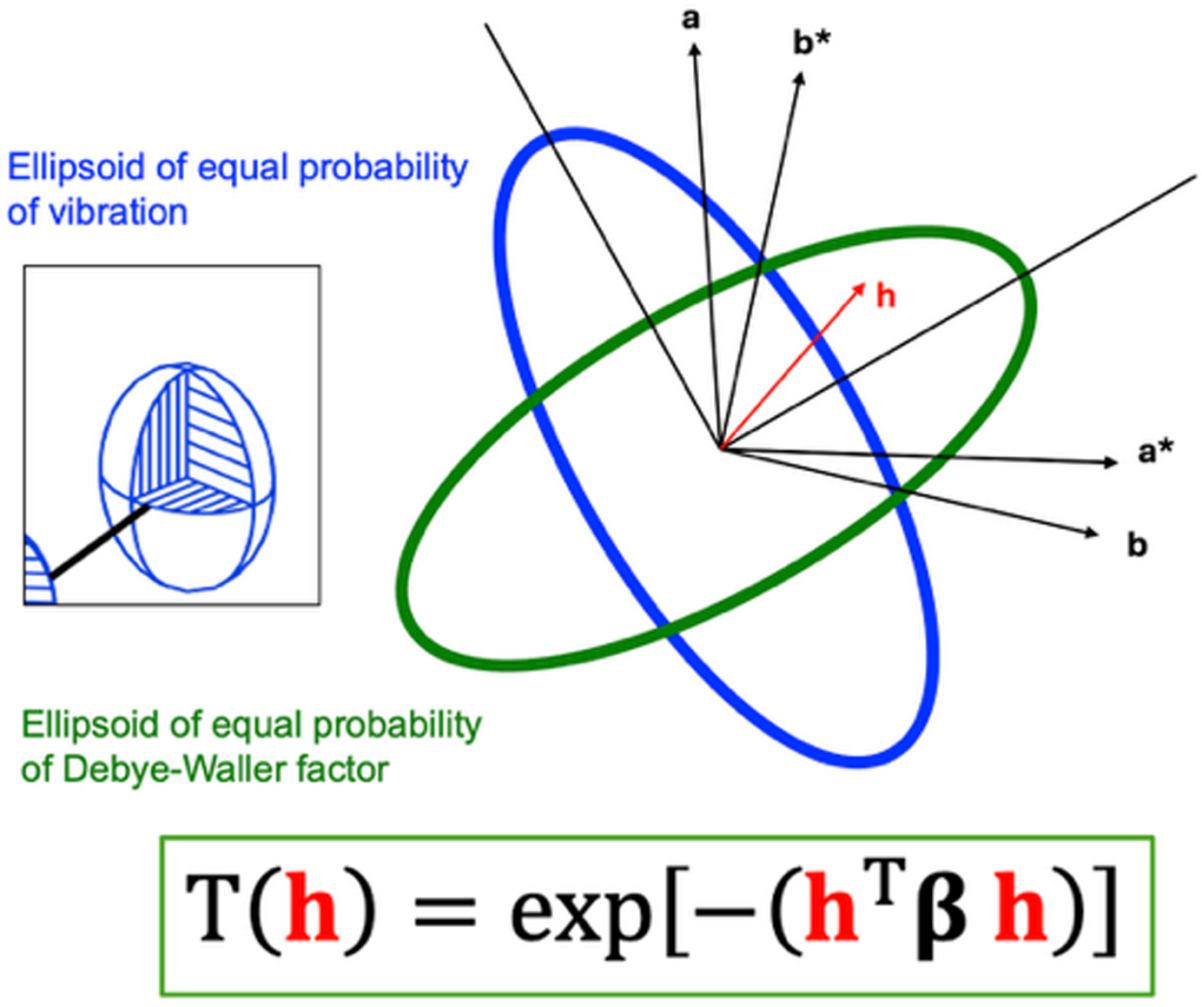
Ellipsoids used in crystallographic illustrations. The 3D equal-probability ellipsoids (shown in the insert) can be understood by considering the 2D example, where ellipses of equal probability can be shown in both direct (blue) and reciprocal (green) space. Illustration inspired by the excellent book by Dunitz (1995 ▸).

In the equation above, the probability density function *p_m_*(**u**) can take any form. In almost all crystallographic models, the choice is to use a 1D or 3D Gaussian function. In fact, it can be shown using the lattice-dynamical theory (see below) that, in the harmonic approximation, this is the correct form of the function (Willis & Pryor, 1975 ▸; chapter 4). The Fourier transform of a 3D Gaussian is conveniently also a Gaussian, and the Debye–Waller factor assumes the following expression:
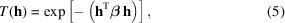
where 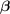 is a symmetrical 3 × 3 matrix with components β^*ij*^. The quantity β^*ij*^ is one of the forms of the anisotropic displacement parameter (ADP). In typical crystallographic software it is more common to use the following form of the displacement parameters:
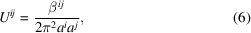
where *a^i^*, *a^j^* are the lengths of the reciprocal lattice vectors. The exact expression for *T*(**h**) varies in the literature, because the **U** tensor can be expressed in different coordinate systems. A very thorough description of these matters can be found in the report of an IUCr Subcommittee on Atomic Displacement Parameter Nomenclature (Trueblood *et al.*, 1996 ▸), and we can also recommend the review by Downs (2000 ▸). The elements of **U** have dimension (length)^2^ and can be associated with the mean-square displacement in the corresponding directions. The 3D Gaussian function defined by **U** can be illustrated by surfaces of constant probability: these are the displacement ellipsoids that are common in crystallographic structure illustrations, see Fig. 1 ▸, where the insert shows the typical 3D surface and we show a 2D example of an equal-probability surface of an anisotropic probability density function (blue) and the corresponding equiprobability contour of the Debye–Waller factor. The larger the mean square displacement (given by the blue surface), the smaller the scattering power of the atom (given by the green surface) in that direction.

There is one overall problem related to the use of atomic displacement parameters. Implicitly, it is assumed that each atom is vibrating independently in the mean field of the surrounding atoms in the crystal. However, it is rather the case that the atoms are interacting, and covalently bound atoms are to a large extent behaving as rigid entities (with a few torsional motions) that vibrate collectively in the crystal.

These phenomena are considered by the theory of lattice dynamics.

## 3. Lattice dynamics

Born and von Kármán introduced the idea of treating the crystal as a periodic array of atoms related by harmonic potentials (springs), which can vibrate collectively. They proposed periodic boundary conditions, meaning the crystal is imagined as repeating infinitely in all directions. This simplification allows for the calculation of vibrational modes and frequencies, which provides an understanding of heat capacity, thermal conductivity, elastic constants and other properties of the crystal. It also provides a direct link to the Debye–Waller factors that we can derive from diffraction measurements.

It is not the purpose of this text to describe the theory of lattice dynamics in full, but we think it makes sense to give a sketch of it, so that we can contrast the lattice-dynamical model with the simpler models that are typically used in crystallography. An in-depth description of lattice dynamics can be found in many textbooks (*e.g.* Willis & Pryor, 1975 ▸; Dove, 1993 ▸). We also recommend Gavezzotti (2007 ▸), which inspired the description below.

The lattice-dynamical approach describes atomic vibrations in terms of travelling waves through the crystal. Each wave – called a phonon – has a particular pattern of motion of atoms. The associated wavelength of these phonons affects the energy – and thus frequency – of the motion. This relation between the wavelength of the phonon and the corresponding frequency can be summarized in a so-called ‘dispersion diagram’. On this diagram, normalized reciprocal space vectors describe the direction of propagation of phonons and how the wavelength of these phonons is related to their frequencies of vibration.

In the language of lattice dynamics, wavevectors expressed in the reciprocal lattice are given the symbol **k**. Each wavevector **k** represents a direction and wavelength λ of a propagating wave. There is an inverse relationship between the length of **k** and the wavelength: |**k**| = 2π/λ. Because of the periodic nature of crystals, it suffices to consider a small fraction of reciprocal space, namely the space from a reciprocal lattice point and half-way to each neighbouring lattice point. This space is called the irreducible Brillouin zone, or simply ‘the Brillouin zone’. In a real 3D crystal, the Brillouin zone is a 3D space. For visualization purposes, the dispersion relations are typically depicted in certain directions of interest of reciprocal space, which are often chosen to be specific high-symmetry directions. These directions are selected because they provide the most information about the vibrational properties of the materials. Most notably, the Γ (Gamma) point is the centre of the Brillouin zone (|**k**| = 0) and represents the long-wavelength limit of the phonon modes, and the *X* point is the boundary of the Brillouin zone along the [100] direction.

In a phonon vibration, atoms in different unit cells show the same motion, but not at the same time. If the wavelength of the phonon is long compared with the lattice dimensions, equivalent atoms in adjacent unit cells will move almost in phase. If the wavelength is short, equivalent atoms – or molecules – in ajacent unit cells will be out of phase.

**Figure 2:**
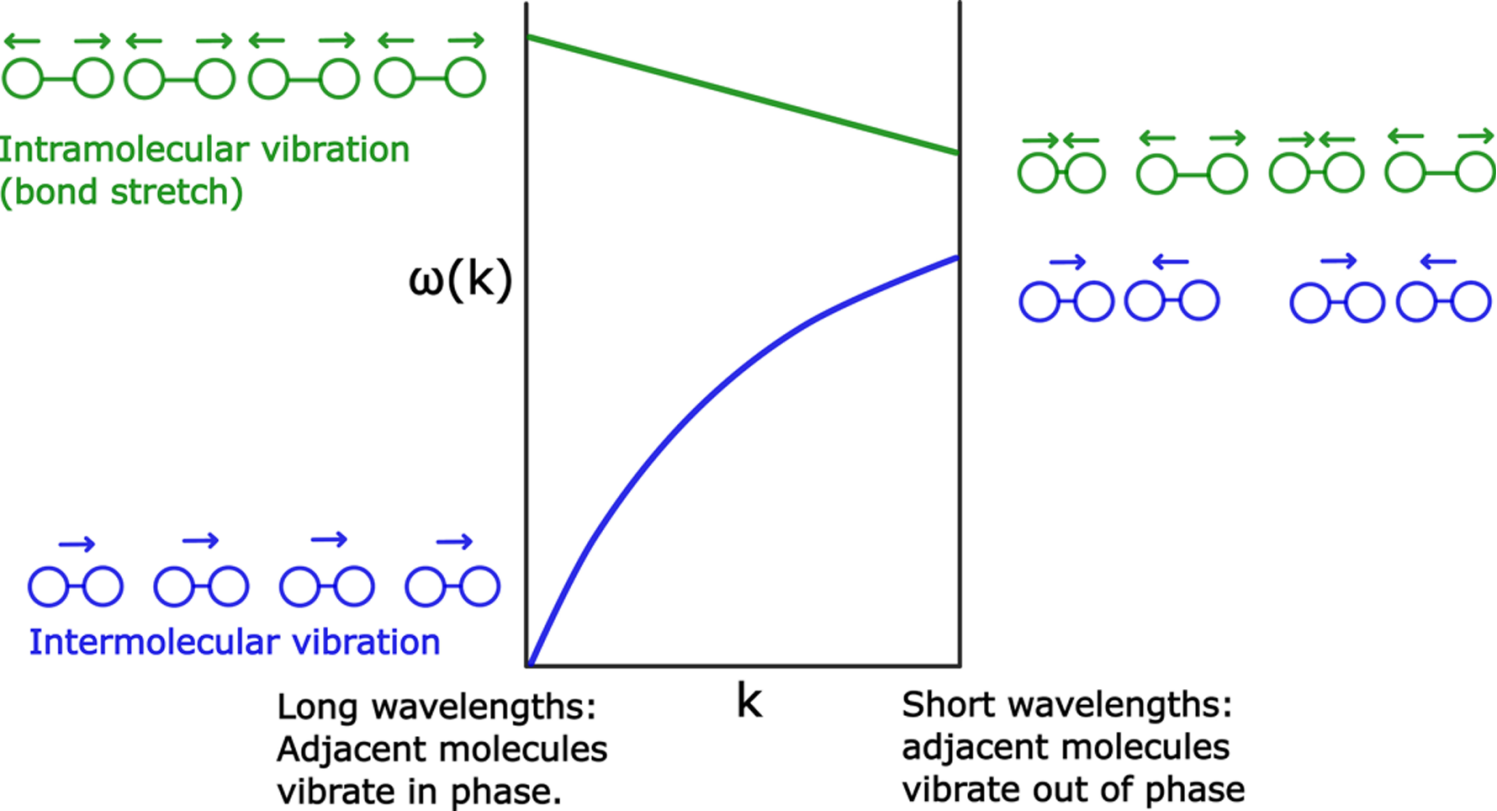
The basic idea of dispersion relations in the theory of lattice dynamics, for a two-atom unit cell.

In order for the reader to grasp the idea of the lattice-dynamical model, we consider a 1D case: consider an endless string of two-atom molecules separated by a lattice spacing (Fig. 2 ▸); the molecules will vibrate along the string, and the movements of the molecules will depend on the neighbouring molecules, but also on next-nearest neighbours and further on to some extent.

In the computational machinery of lattice dynamics, the force constants between each and every atom have to be calculated. Of course, if we consider an endless string, this would sum up to an infinite number of calculations; however, at longer distances the forces become insignificant, and it is often sufficient to calculate forces in a small supercell.

In Fig. 2 ▸ we consider two typical types of motion. The first is the intermolecular vibration, where rigid molecules interact with each other (shown in blue). On the left side of the dispersion diagram, |**k**| is small and the wavelength of the vibration is very large. This implies that the molecules vibrate in phase, and the frequency ω of the vibration (which is proportional to the energy) is also very small, going towards zero as the wavelength goes towards infinity. Following this (blue) phonon branch towards the border of the Brillouin zone (right side), we have a short wavelength, where adjacent molecules are out of phase: they bump in to each other. This corresponds to a much larger energy of the phonon, which is reflected in the larger frequency of the vibration.

**Figure 3:**
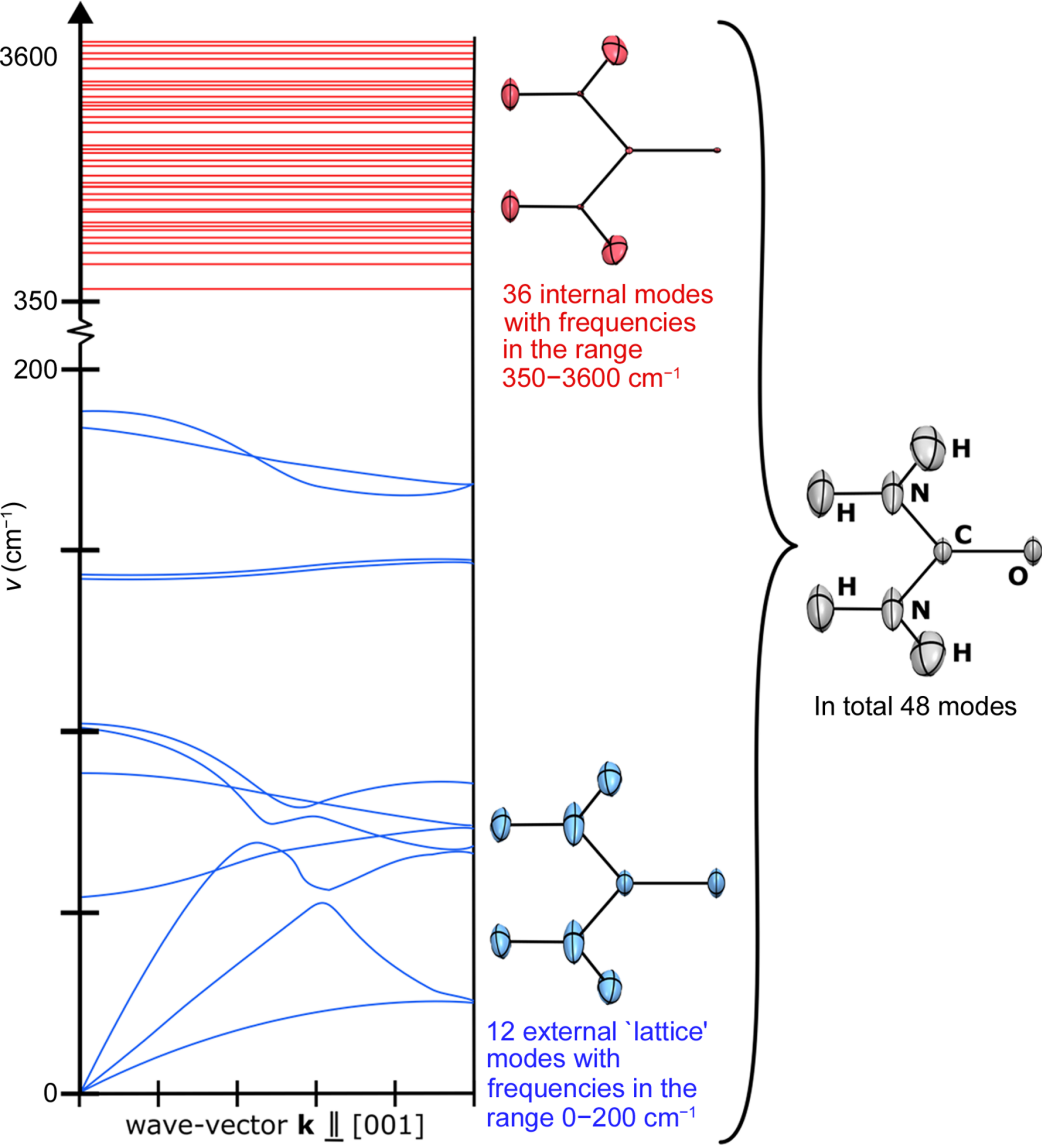
Dispersion curves of the urea crystal in the [001] direction of reciprocal space, as well as calculated displacement ellipsoids (50%) corresponding to 123 K, based on *ab initio* calculations from Madsen *et al.* (2013 ▸). Blue and red indicate the low-frequency ‘external’ phonons and ‘internal’ phonons, respectively. The two domains of motion contribute differently to the displacements, as seen in the ellipsoid drawings in blue and red, and sum up to the ellipsoids depicted in greyscale. Published dispersion curves typically cover several directions in *k*-space, and would be more complicated than what we depict here.

The second vibration (green) that we consider is the intramolecular bond stretch. The intramolecular vibrations typically have higher frequencies than the intermolecular ones. When we contrast the in-phase vibrations (left side of the diagram) with the out-of-phase diagram (right side), we see that the intermolecular distances are shorter, and thus the energy is higher when the molecules are vibrating in phase. The dispersion curve is therefore declining as |**k**| becomes higher. In a real 3D molecular system there are many more dispersion curves (see an example for urea in Fig. 3 ▸). If the molecules are flexible, there will be no sharp delimiter between the low-frequency intermolecular vibrations and the high-frequency intramolecular ones. Rather, there will be an intermediate region in the dispersion diagram which consists of modes that combine inter- and intramolecular vibrations.

The basic behaviour of phonons, as laid out in the lattice-dynamical theory, has proven to be a useful model that corresponds well with experimental results. In particular, dispersion relations such as the one sketched in Fig. 2 ▸ can be measured using inelastic neutron scattering experiments on single crystals. This technique has been an important tool for studying extended solids, such as minerals (Chaplot *et al.*, 2002 ▸). These experiments are time consuming and require very large crystals (some cubic centimetres), and it is no wonder that relatively few dispersion curves have been mapped out. In the case of molecular crystals, only a handful of systems have been explored, and these systems consist of very small molecules in lattices of high symmetry (Micu *et al.*, 1995 ▸; Pawley, 1969 ▸; Schatschneider *et al.*, 2012 ▸; Chaplot *et al.*, 1983 ▸; Lefebvre *et al.*, 1975 ▸; Dolling & Powell, 1970 ▸; Natkaniec *et al.*, 1980 ▸; Dove *et al.*, 1989 ▸).

A great deal of information about vibrational modes can also be obtained from Raman and infrared spectroscopies. These techniques provide information on the frequencies of phonons in the long-wavelength limit (the left edge of the diagram in Fig. 2 ▸, which is called the Γ point).

Lattice-dynamical theory relies on the harmonic approximation. This implies that phenomena such as thermal expansion cannot be explained based on this model. Lattice-dynamical models moving beyond the harmonic approximation are being proposed (Monacelli *et al.*, 2021 ▸; Castellano *et al.*, 2023 ▸).

## 4. Calculation of Debye–Waller factors from theory

If a full lattice-dynamical model has been constructed – *i.e.* a description of the lattice vibrations (called normal modes) and their frequencies, covering all areas of the Brillouin zone in a dense grid – then it is possible to calculate the atomic Debye–Waller factors by summing mean square displacements from all possible normal modes. Such a model can be obtained in a number of ways, as outlined in the following sections.

### 4.1. Force fields fitted against experimental dispersion curves

One approach is to fit a model of interatomic forces against the dispersion curves obtained from inelastic neutron scattering and Raman spectroscopy. This was done for urotropine (Willis & Howard, 1975 ▸) and for silicon (Flensburg & Stewart, 1999 ▸). In both cases, the comparison between the Debye–Waller factors obtained from diffraction measurements and the lattice-dynamical approach showed a very good agreement. These results testify that in these cases, the ADPs that we derive from X-ray and neutron crystallography do correspond to the mean-square displacements of the atoms in the crystal, and are only biased by other effects (see Section 5) to a lesser extent.

### 4.2. *Ab initio* and force-field calculations

The lattice-dynamical model has been used to calculate the Debye–Waller factors in a number of studies. Pioneering work in the late 1970s and 1980s by Gramaccioli and co-workers was done on molecular crystals using empirical force fields (Gramaccioli *et al.*, 1982 ▸; Gramaccioli & Filippini, 1983 ▸). These studies showed a reasonable agreement with experimental Debye–Waller factors from neutron diffraction studies. In recent years, it has become feasible to derive the force constants from *ab initio* calculations to set up the lattice-dynamical model. One approach that has been used quite extensively is to perform periodic calculations, either using atom-centred basis sets (Madsen *et al.*, 2013 ▸) or plane-wave calculations (Deringer *et al.*, 2014 ▸; George *et al.*, 2015 ▸, 2016 ▸). A computationally faster alternative is to use so-called *ONIOM* calculations, which describe central atoms at a high level of theory and atoms in the perimeter of the system at a lower level (Dittrich *et al.*, 2012 ▸).

In such *ab initio* calculations, it is often found that the frequencies of intramolecular vibrations match very well with information from Raman and IR spectroscopy (Scott & Radom, 1996 ▸). However, the ‘softer’ vibrations that are dominated by intermolecular motion, and also combinations of inter- and intramolecular vibrations, are much more difficult to calculate. This is because the potential energy hypersurface that determines the frequencies of these vibrations is based on quite weak interactions, including dispersion forces that are difficult to assess accurately using density-functional theory (DFT) calculations. The majority of the contribution to the ADPs comes from the low-frequency modes, as can be seen in Fig. 3 ▸, which illustrates some dispersion curves and the corresponding displacement ellipsoids for the urea crystal. Nevertheless, the resulting calculated ADPs are often found to be in reasonable agreement with the experimentally determined ADPs. To consider the temperature-dependent lattice expansion, one can apply the so-called quasi-harmonic approximation, where the harmonically approximated forces are calculated at different (experimentally determined) lattice dimensions corresponding to different temperatures. This approach considerably improves the agreement of measured and calculated ADPs at higher temperatures (George *et al.*, 2017 ▸).

Most software packages that perform periodic DFT calculations can compute a lattice-dynamical model. Some of these programs can also compute the ADPs [*e.g.* the *CRYSTAL* program (Erba *et al.*, 2023 ▸)], but there is also dedicated auxiliary software that can perform the task, one notable example being the program *phonopy* (Togo *et al.*, 2023 ▸), which can interface to many different periodic *ab initio* programs.

### 4.3. Debye–Waller factors from molecular dynamics simulations

The lattice-dynamical approaches mentioned above typically employ the harmonic approximation, which is sufficient for calculating ADPs. However anharmonic effects are often seen in high-resolution crystallographic studies (see further in Section 6). In this context, molecular dynamics simulations can provide an alternative view beyond the harmonic approximation. Reilly *et al.* (2007 ▸, 2011 ▸) used such *ab initio* molecular dynamics simulations in order to come up with alternative functions to describe atomic motion in crystallographic models providing curvilinear motion which cannot be obtained with the standard harmonic approach.

Molecular dynamics simulations are more often based on empirical force fields. This implies that the calculations are orders of magnitude faster than the *ab initio* based approaches. However, the results are very dependent on the applied force field, and this approach tends to underestimate the magnitude of ADPs compared with the experimental results (Nemkevich *et al.*, 2010 ▸). A promising alternative can be to use machine learning to derive force fields from *ab initio* calculations. This approach is faster than *ab initio* molecular dynamics and more accurate than classical force fields (Liu *et al.*, 2021 ▸).

## 5. Anisotropic displacement parameters from diffraction experiments

As mentioned in the introduction, Debye–Waller factors can be obtained not only from X-ray diffraction measurements, but also from neutron and electron diffraction measurements.

ADPs can be affected by many factors. Some of them are obvious and arise from the physical meaning of the ADPs, *e.g.* temperature (higher temperature gives larger ADPs), atomic mass (heavier atoms give smaller ADPs) and the APDs must obey the crystal symmetry.

While it is common for articles reporting new structures to include figures depicting displacement ellipsoids, the accurate determination of these parameters is neither a straightforward nor a trivial task. In the following section, we provide a detailed explanation of the underlying reasons. We will first focus on single-crystal X-ray diffraction measurements, and then discuss neutron diffraction.

The intensity of a Bragg peak is given by the equation
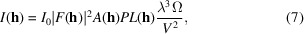
where *I*_0_ is the intensity of the incident beam; |*F*_**h**_|^2^ is the squared modulus of the structure factor for reflection **h**, which includes the Debye–Waller factor; *A* is the absorption coefficient; *P* is the polarization correction; *L* is the Lorentz factor; λ is the radiation wavelength; Ω is the volume of a crystal; and *V* is the unit-cell volume.

It is immediately apparent that inaccurate modelling of density or issues with the absorption coefficient can lead to unreliable ADPs. Any problems arising from an incorrect structural model, improper electron density description, absorption corrections or other experimental difficulties will likewise impact the accuracy of the ADPs. Consequently, ADPs are frequently regarded as a catch-all for various experimental errors.

### 5.1. Disorder or thermal motion?

One should keep in mind that ADPs are ‘displacement factors’ not just temperature factors. Displacement of atoms from their positions in the crystal might be related not only to thermal vibrations and motion of atoms, but also to any type of disorder. Configurational disorders, however small, will affect ADPs. In some cases, it might be difficult to distinguish between disorder and thermal motion – multi-temperature measurements can help [ADPs that appear elongated at higher temperatures might model several atomic sites, which can be revealed when the thermal motion is diminished at lower temperatures (Bürgi, 2000 ▸), or diffraction can be complemented by other techniques such as solid-state NMR (Moran *et al.*, 2017 ▸)]. Occupational disorders, modulations and thermal diffuse scattering (Wahlberg & Madsen, 2017 ▸) will also affect ADPs.

### 5.2. Factors related to experiment: data collection strategy

It is important to be aware that the strategy used during single-crystal X-ray diffraction measurement is crucial for obtaining accurate ADPs. Data collection resolution and completeness strongly affect the ADPs. When the resolution is not high enough, bonding density might not be separated from ADPs accurately and the ADPs might be too large, especially in the bond direction. It was shown by Parsons with leverage analysis in an analysis of diffraction data for l-alanine that data above sin(θ)/λ = 0.6 Å^−1^ are most influential for the ADPs (Parsons *et al.*, 2012 ▸). Application of modern quantum crystallography techniques during refinement might solve the problem of deconvolution of bonding density from ADPs as we discuss further in Section 5.4.

It is much more complicated to overcome problems related to data completeness, which are often encountered in the case of complicated experimental setups such as high pressure, humidity cells or furnaces, as well as for collection of Laue neutron diffraction data and electron diffraction data. It is well known (however not studied systematically yet) that a missing cusp of data leads to elongation of ADPs along the direction where the data are missing. Obviously, in the case of all types of experiments it is important to try to obtain the best possible completeness. In the case of high-pressure data, it can be done by orienting the crystal properly in the diamond anvil cell (DAC) (Tchoń & Makal, 2021 ▸). Another option to improve completeness is to merge datasets from different crystals, although this may also be problematic (*e.g.* in the case of electron diffraction data), where dynamical scattering effects will be different for every crystal. Usually in the case of low-symmetry compounds even with proper orientation of the crystal in a DAC it might be difficult to get full completeness. Thus, during the refinement of such incomplete data, in many cases constraints and restraints on ADPs must be imposed. Note that ADPs may also be affected by radiation damage [as shown by McMonagle *et al.* (2024 ▸)].

### 5.3. Factors related to data treatment

The correct treatment of data is crucial for obtaining accurate ADPs. In the case of compounds containing heavy and strongly absorbing elements, an absorption correction should be applied. It was recently shown that, for strongly absorbing atoms, ADPs computed using DFT calculations are in better agreement with those obtained from the refinement of synchrotron data (smaller crystals, shorter wavelength, less absorption) than from in-house measurements (Mroz *et al.*, 2020 ▸). The impact of refining an extinction coefficient and the use of a weighting scheme for the observed intentities during refinement was investigated in a study of low albite (Armbruster *et al.*, 1990 ▸), where differences of about 0.01 Å^2^ were observed depending on the use of an extinction correction and a weighting scheme of 1/σ or no weights. Similar conclusions were drawn by Capelli and co-workers (Bürgi *et al.*, 2000 ▸); errors in the ADPs might be the result of insufficient extinction correction in the diffraction data.

### 5.4. Factors related to refinement

Pioneers in experimental charge density studies, Philip Coppens and Tibor Koritzansky have stated that ‘no reasonable estimate of the charge density parameters can be obtained without an adequate description of the thermal motion’ (Koritsanszky & Coppens, 2001 ▸). Obviously, this kind of relation is mutual: without an adequate description of the electron density, it is not possible to accurately characterize thermal motion. Therefore, to get the best possible thermal motion description, it is crucial to use the best possible density models. At present the most frequently applied models which take the asphericity of the electron density into account are the TAAM (transferable aspherical atom model) (Dominiak *et al.*, 2007 ▸; Jarzembska & Dominiak, 2012 ▸) and the HAR (Hirshfeld atom refinement) (Jayatilaka & Dittrich, 2008 ▸; Capelli *et al.*, 2014 ▸). It was shown by Sanjuan-Szklarz *et al.* (2016 ▸) that by application of TAAM or HAR one can improve the deconvolution of thermal motion and electron density even for low-resolution data.

HAR and TAAM refinements can easily be conducted in *Olex2* (Dolomanov *et al.*, 2009 ▸) using *NoSpherA2* (Kleemiss *et al.*, 2021 ▸), and we would advocate this type of refinement for standard structure solution in order to obtain more accurate atomic coordinates as well as ADPs.

It has become possible to use aspherical density models even for disordered structures. In the presence of disorder, it is not uncommon for the ADPs to appear elongated. This could indicate that the refinement is not adequately capturing the complexity of the disorder, or that the disorder is too subtle or hidden by symmetry (Bürgi & Capelli, 2003 ▸). If the disorder cannot be described properly by refining multiple atomic sites, it may be necessary to apply constraints or restraints to the ADPs during the refinement process.

Another important consideration during the refinement process is whether a harmonic model sufficiently captures the motion of the atoms, or if it would be more appropriate to employ models that account for anharmonic motion, as we will discuss Section 6.

### 5.5. ADPs from single-crystal neutron diffraction

In the case of neutron diffraction, radiation is scattered not by electron density but by nuclei. In contrast to X-ray atomic scattering factors, scattering lengths for neutron diffraction do not decrease with sin(θ)/λ and are not dependent on the number of electrons or protons. Therefore, single-crystal neutron diffraction offers a unique opportunity to obtain accurate positions and atomic displacements for hydrogen atoms. This advantage is particularly important in the case of strong hydrogen bonds (Cowan *et al.*, 2005 ▸; Wilson & Thomas, 2005 ▸) or other exotic *X*—H bonds. Additionally, hydrogen atom positions and ADPs obtained from neutron diffraction can be applied when performing multipolar refinement for accurate experimental charge density determination (*e.g.* Sovago *et al.*, 2014 ▸). Moreover, joint X-ray–neutron refinement against two datasets can be conducted (Liebschner *et al.*, 2023 ▸; Coppens *et al.*, 1981 ▸). Interesting conclusions can be made by comparing ADPs of non-hydrogen atoms obtained for the same structures by neutron and X-ray measurements at the same temperature. It appears that ADPs from these two different diffraction techniques may differ significantly. This phenomenon was investigated by Blessing (1995 ▸), who explained that differences in ADPs obtained by various diffraction techniques might arise due to different experimental conditions (*e.g.* slight variations in temperature). Additionally, as neutron and X-ray diffraction use different types of radiation and measure different crystal sizes (larger for neutron diffraction and relatively smaller for X-rays), factors such as absorption, extinction and multiple scattering – if not properly accounted for – can contribute to discrepancies between neutron and X-ray ADPs. Thus, Blessing (1995 ▸) proposed that if hydrogen atom ADPs from neutron diffraction are to be used during X-ray refinement, the non-hydrogen ADPs should first be compared. Based on this comparison, appropriate scaling factors for hydrogen atom ADPs should be applied. This comparison and scaling can be performed using the *UIJXN* program (Blessing, 1995 ▸). Later, an interesting comparison of ADPs from neutron and X-ray diffraction was conducted by Morgenroth *et al.* (2008 ▸), who analysed ADPs from neutron and X-ray diffraction for seventeen different structures [see Table 2 in Morgenroth *et al.* (2008 ▸)]. Their study found that an outstanding agreement was achieved for extremely simple crystals, such as metallic Be. However, for molecular crystals, deviations between neutron and X-ray ADPs were larger. Increasing the temperature from helium levels (∼ 10 K) to nitro­gen levels (∼ 100 K) appeared to worsen the agreement between X-ray and neutron ADPs. Nevertheless, it is possible to obtain extremely good agreement between neutrons and X-rays.

Unfortunately, although there has been progress in single-crystal neutron diffraction, it still requires larger single crystals than X-ray diffraction, which are sometimes impossible to grow.

Debye–Waller factors can be obtained not only from single-crystal X-ray or single-crystal neutron diffraction, but also from powder diffraction via Rietveld refinement. Many studies in which accurate Debye–Waller factors were obtained by neutron powder diffraction for inorganic systems were conducted (*e.g.* Tilli *et al.*, 1980 ▸; Mohanlal, 1979 ▸; Lawson *et al.*, 1992 ▸), in some cases thermal evolution of Debye–Waller factors was investigated (*e.g.* Vidal & Vidal-Valat, 1986 ▸). Recently, it was shown that with current development of quantum chemistry methods, for rubidium and caesium ureate, it is possible to refine against the experimental powder diffraction neutron data using starting parameters from *ab initio* simulations of atomic positions and ADPs (Sterri *et al.*, 2016 ▸).

We emphasize that the availability of neutron sources is limited, as they are typically found only at specialized research facilities. Producing a neutron beam requires either a reactor or a spallation source, making access to measurements more challenging and time consuming. However, when neutron diffraction data can be obtained, they are highly beneficial, as they provide information that is absent in X-ray diffraction data (*e.g.* the positions and ADPs of hydrogen atoms, magnetic properties).

## 6. Harmonic versus anharmonic approximation

**Figure 4:**
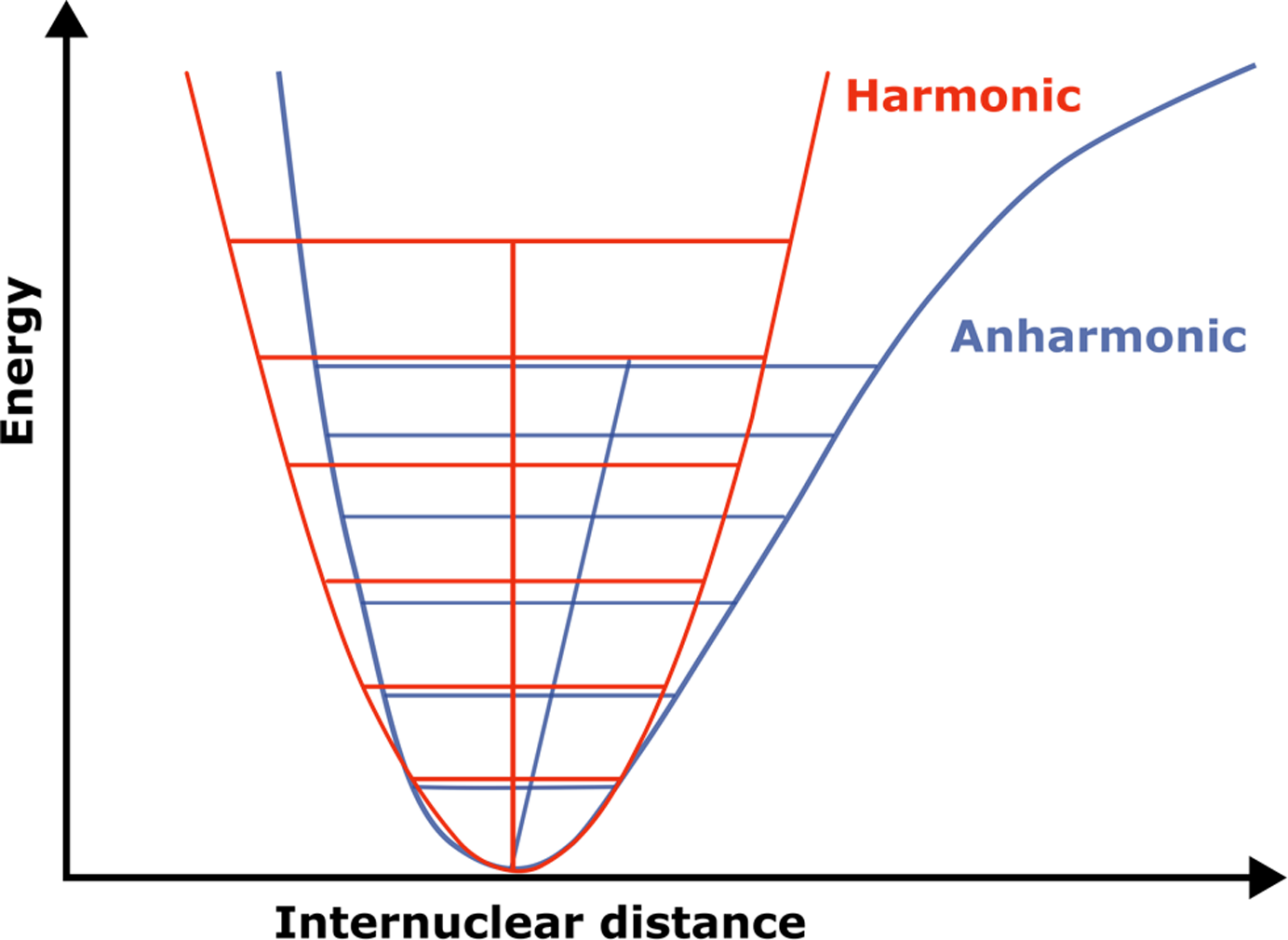
Harmonic versus anharmonic potential. In the anharmonic case, the average internuclear distance changes as a function of temperature, when more energy levels are populated.

Vibrations of atoms are usually described by means of the harmonic approximation; it is a fast and simple model. Unfortunately, the potential energy surface in a crystal is often more complex. Many physical properties would not exist and cannot be correctly described within the harmonic approximation, *e.g.* thermal expansion. In a harmonic potential (Fig. 4 ▸), a rise in temperature will allow more vibrational modes to be populated, but the mean value of the interatomic distance remains the same. This implies that distances between atoms and, as a consequence, unit-cell parameters, do not change as a function of temperature within this approximation. In contrast, when anharmonicity is considered the potential curve has a nonsymmetric shape, and one can immediately see that the mean values of internuclear distances are increasing with the temperature because higher energy levels are populated.

Beyond the harmonic approximation the probability density function, *p_k_*(**u**), will not be well described by a 3D Gaussian function. This means that ADPs, which are purely harmonic, will not be able to model anharmonic effects. This might be manifested in unmodelled residual density near atoms. The residual density will have a characteristic shashlik-like pattern.

The first thorough theoretical investigations into the effects of anharmonicity on atomic displacements were conducted in the early 1960s by Krivoglaz & Tokhonova and Maradudin & Flinn (1963 ▸). The first report of anharmonic structure refinement came when Burns *et al.* (1963 ▸) applied it to single-crystal neutron diffraction data of XeF_4_. A large range of studies related to anharmonicity were carried out and summarized by Kuhs (1988 ▸, 1992 ▸). In the cited contributions, Kuhs described the methods which can be applied to model anharmonic motion of atoms, *i.e.* deconvolution of the thermal factors into Gram–Charlier (Johnson & Levy, 1974 ▸) or Edgeworth series (Johnson, 1969 ▸), and the OPP (one particle potential) formalism. In the most frequently applied approach, Gram–Charlier coefficients are used as parameters during refinement. The harmonic Debye–Waller factor is amended so that the full Debye–Waller factor is expressed with the following formula,
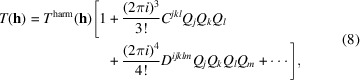
where 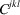 and 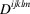 are the Gram–Charlier third- and fourth-order anharmonic terms, and *Q_j_* = *h_j_*/2π. Here we use Einstein’s summation rule over repeated indices, and *j*, *k*, *l*, *m*, *n* = 1, 2, 3, respectively, are the Miller indices.

A selection of graphical representations of density modulations due to higher-order terms in the Gram–Charlier series expansion of a Gaussian atomic probability density function are provided in *International tables for crystallography* Volume D (Kuhs, 2013 ▸).

The use of Gram–Charlier parameters comes with a substantial increase in the number of refined parameters. The use of third-order terms increases the number of parameters per atom to 25, and the number of data required for having a meaningful refinement is high. According to Kuhs (1992 ▸), many attempts to model anharmonicity have failed because the dataset was not measured at sufficiently high resolution, rather than because of deficiencies in the models or the quality of the data. Kuhs formulated rules for the diffraction measurements which should be obeyed in order to meaningfully refine anharmonic motion. In many cases, where the data resolution is limited, it might be more meaningful to consider a model of curvilinear nature, as discussed in Section 4.3.

Refinement of anharmonic motion was carried out even before models which consider asphericity of the electron density distribution were developed. Therefore, when unexplained residual density appeared in the model, it was hard to distinguish whether it came from undescribed anharmonic motion or whether it was just a bias of the spherical independent atom model.

Thus, many studies related to anharmonicity have been done with neutron diffraction, which probes the nuclei, and therefore are not prone to these deconvolution problems (Volkov *et al.*, 2023 ▸). Development of models which account for the asphericity of the electron density allowed for detection of anharmonicity in many systems using high-resolution X-ray diffraction. The first studies which combined anharmonic motion treatment with multipolar formalism were done by the Philip Coppens group (Mallinson *et al.*, 1988 ▸). Restori & Schwarzenbach (1996 ▸) showed that multi-temperature measurements are crucial for unambiguously separating effects related to anharmonic atomic motion and chemical bonding. Meindl *et al.* (2010 ▸) analysed how the residual density distribution as well as the location and strength of valence-shell charge concentrations change as a consequence of neglected anharmonic effects. In a very thorough contribution, Herbst-Irmer *et al.* (2013 ▸) analysed multiple datasets across varying temperatures and demonstrated that the anharmonic model in the case of 9-di­phenyl­thio­phosphinoylanthracene outperformed the disorder model. They found that the refined multipole parameters were affected when the anharmonic motion was not adequately addressed (Herbst-Irmer *et al.*, 2013 ▸). This study underscored the significance of accurately identifying and managing anharmonic motion. Later on, anharmonic motion was modelled and analysed in many experimental charge density studies (Destro *et al.*, 2021 ▸; Tidey *et al.*, 2017 ▸; Hübschle *et al.*, 2018 ▸; Jarzembska *et al.*, 2015 ▸, 2017 ▸). In some recent contributions it was shown that anharmonic motion can be refined not only in the case where the charge density is described by a multipole model but also when HAR and X-ray wavefunction fitting is employed (Woinska *et al.*, 2019 ▸).

The Gram–Charlier formalism focuses on deviations of the probability density function due to anharmonicity. However, as clearly illustrated in Fig. 4 ▸, the harmonic approximation fails to account for thermal expansion, and the potential energy curve diverges significantly from the harmonic form, particularly at higher temperatures. This discrepancy affects the evolution of atomic displacement parameters with temperature, and should be considered in the analysis of multi-temperature data, as has been done by Bürgi *et al.* (2000 ▸). We discuss their approach in Section 8. For more information related to anharmonicity, an excellent mini-review is recommended (Volkov *et al.*, 2023 ▸).

## 7. Validation of ADPs

Once the refinement process is completed and has converged, it is crucial to conduct a thorough analysis of the refinement statistics and residual density. In addition, verifying the atomic displacement parameters is essential. A visual inspection of the atomic displacement parameters can often reveal important insights into the accuracy of the structure, such as identifying misassigned atoms and other potential issues. For instance, if the atomic displacement parameters are oriented consistently in one direction, this may indicate problems with absorption correction. Moreover, small amounts of disorder within the structure can often be detected through visual examination of the atomic displacement parameters.

In addition to a visual inspection of atomic displacement parameters, there are several tests that can be performed to verify their reliability. One such test that is routinely conducted as part of the *checkCIF* procedure is the Hirshfeld rigid-bond test (Hirshfeld, 1976 ▸). The rigid-bond criterion posits that the mean-square displacement amplitudes of atoms connected by a covalent bond are equal along the direction of that bond. This test requires the calculation of the vibration amplitudes in the direction of the bonds.

**Figure 5:**
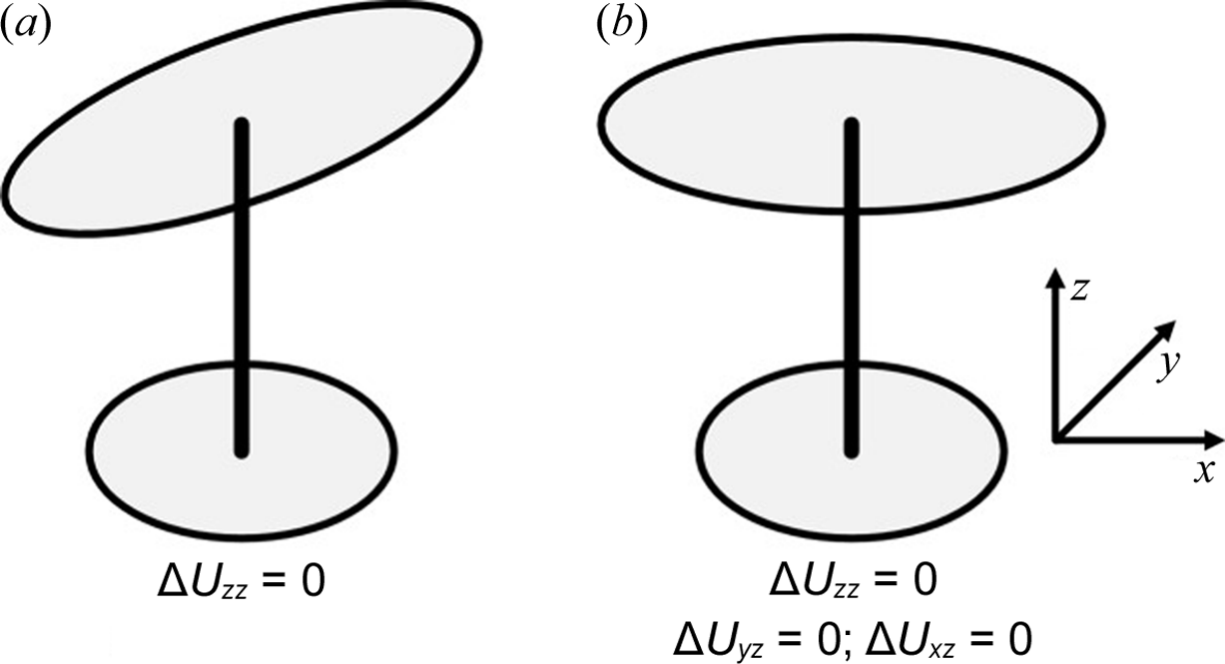
The rigid-bond criterion is satisfied by both (*a*) and (*b*), but only (*b*) fulfils the enhanced rigid-bond restraint, as defined by Thorn *et al.* (2012 ▸), from where the figure originates.

In addition to being used solely for validation purposes, restraints on atomic displacement parameters can also be established based on the principles of rigid-bond assumptions. Rollett (1970 ▸) applied rigid-bond restraints in the least-squares refinement of crystal structures by means of additional observational equations. This approach was later applied in *SHELXL* (Sheldrick, 2008 ▸) and *REFMAC* (Murshudov *et al.*, 2011 ▸). Subsequently, Thorn *et al.* (2012 ▸) reformulated the Hirshfeld rigid-bond condition, so that the relative motion of the two atoms is required to be perpendicular to the bond, a so-called enhanced rigid-bond restraint) – the different rigid bonds are depicted in Fig. 5 ▸.

## 8. Analysis of ADPs: rigid-body analysis and beyond

The rigid-bond tests and constraints mentioned in the previous section try to impose physically meaningful restraints on the ADPs. In a similar manner, rigid-body analysis is an attempt to analyse the atomic mean square displacements of a molecule as if the molecule was vibrating as a rigid unit, vibrating in the mean field of the surrounding molecules in the crystal. This type of analysis can be applied to ADPs coming from any diffraction technique. Cruickshank (1956*e* ▸) showed the connection between displacement and frequency of each mode of vibration and developed the first analysis of ADPs in terms of rigid-body vibrations.

Following the pioneering work of Cruickshank (1956*c* ▸,*b* ▸), researchers have analysed the ADPs as if they originated from collective motion with a considerable amount of success. The most well known model is the translation/libration/screw (TLS) model developed by Schomaker & Trueblood (1968 ▸), which has been used in numerous applications. The TLS approach can be extended to include ‘attached rigid groups’, and this allows for characterization of internal degrees of freedom that have been used derive force constants of such vibrational modes (Trueblood & Dunitz, 1983 ▸; Dunitz *et al.*, 1988 ▸). One prominent objective of applying the rigid-body model has been to obtain libration corrections to the inter­atomic distances: as first noted by Cruickshank (1956*a* ▸), librational motion of molecules causes the refined atomic positions to be slightly displaced from the true positions towards the rotation axes.

The ADPs do not contain information about the correlation of motion between different atoms; however, since the amplitudes of the vibrational modes depend differently on the temperature, multi-temperature experiments can recover part of this correlation, as shown by Bürgi, Capelli and co-workers (Bürgi & Capelli, 2000 ▸; Capelli *et al.*, 2000 ▸).

A range of computer programs have been developed to perform rigid-body analysis, either using the TLS formalism [*PLATON* (Spek, 1990 ▸) and *THMA11* (Schomaker & Trueblood, 1968 ▸)] or related models [*EKRT* (He & Craven, 1985 ▸, 1993 ▸)].

## 9. The special case of hydrogen atoms

Due to the relatively low scattering power of hydrogen atoms (which arises from the lack of any core electron density), it is difficult to define the positions and thermal parameters for hydrogen atoms using X-ray diffraction data alone. Therefore, for a long time constraints and restraints on both position and isotropic displacement parameters (most commonly elongation of *X*—H bond lengths to standard neutron values and the ‘riding’ approximation) were used during refinements. Current quantum crystallography methods like HAR and TAAM enable us to precisely find positions of hydrogen atoms, even in the case of strong hydrogen bonds or for hydrogen atoms bonded to transition metals. Unfortunately, hydrogen atom ADPs purely from diffraction data are difficult to capture even with HAR – in many cases they are elongated in one direction, sometimes non-positive definite. Despite this obstacle, the positions of hydrogen atoms can be found accurately, even when using an isotropic displacement parameters for the hydrogen atoms. On the contrary, for more accurate calculations of properties of charge density distribution, particularly when employing a multipole model or refining the wavefunction constrained by X-ray data (Jayatilaka & Grimwood, 2001 ▸), it is essential to correct the *X*—H bond distances and apply ADPs for hydrogen atoms during the refinement process (Hoser *et al.*, 2009 ▸; Madsen *et al.*, 2004 ▸; Malaspina *et al.*, 2020 ▸). Hydrogen ADPs can be obtained via theoretical calculations, or from the *SHADE* server or normal-mode refinement, as described below.

### 9.1. The *SHADE* approach

It is possible to analyse the vibrational motion of hydrogen atoms in a similar vein as the statistical analysis of *X*—H bond lengths derived from neutron diffraction studies found in *International tables for crystallography* Volume C (Allen *et al.*, 2006 ▸). When the total atomic mean square displacement tensor *U* has been determined from neutron diffraction experiments, and the rigid molecular motion *U*_rigid_ has been determined from a rigid-body analysis of the non-hydrogen ADPs, it becomes possible to get an estimate of the internal motion of the hydrogen atoms:
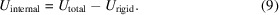
It was noted by Johnson (1970 ▸) that the mean square displacements derived from *U*_internal_ of hydrogen atoms were in good agreement with spectroscopic information, showing systematic trends corresponding to the functional group that hydrogen was part of. Similar observations were made by Craven and co-workers in the analysis of several systems (Gao *et al.*, 1994 ▸; Kampermann *et al.*, 1995 ▸; Luo *et al.*, 1996 ▸; Weber *et al.*, 1991 ▸). The internal torsional motion of a range of librating groups – including methyl, carboxyl and amino – was also thoroughly investigated by Trueblood & Dunitz (1983 ▸) based on more than 125 neutron diffraction studies of molecular crystals from the literature.

Inspired by these results, we analysed a range of neutron structures found in the literature, and the estimates of internal motion were collected in a ‘library’ and later improved and enhanced with more statistical material (Madsen *et al.*, 2003 ▸; Munshi *et al.*, 2008 ▸). The present *SHADE2* library provides mean values of internal stretch modes as well as in-plane and out-of-plane bending modes for a range of chemical groups involving hydrogen bound to C, N and O. The library forms the basis for assigning ADPs to hydrogen atoms in the *SHADE* server (Madsen, 2006 ▸), which allows users to submit a CIF containing the atomic coordinates and the ADPs of the non-hydrogen atoms. The server performs a TLS analysis using the *THMA11* program and combines the rigid-body motion with the internal motion obtained from analysis of neutron diffraction data. The *SHADE* server is available at http://crystallography.if.ku.dk.

**Figure 6:**
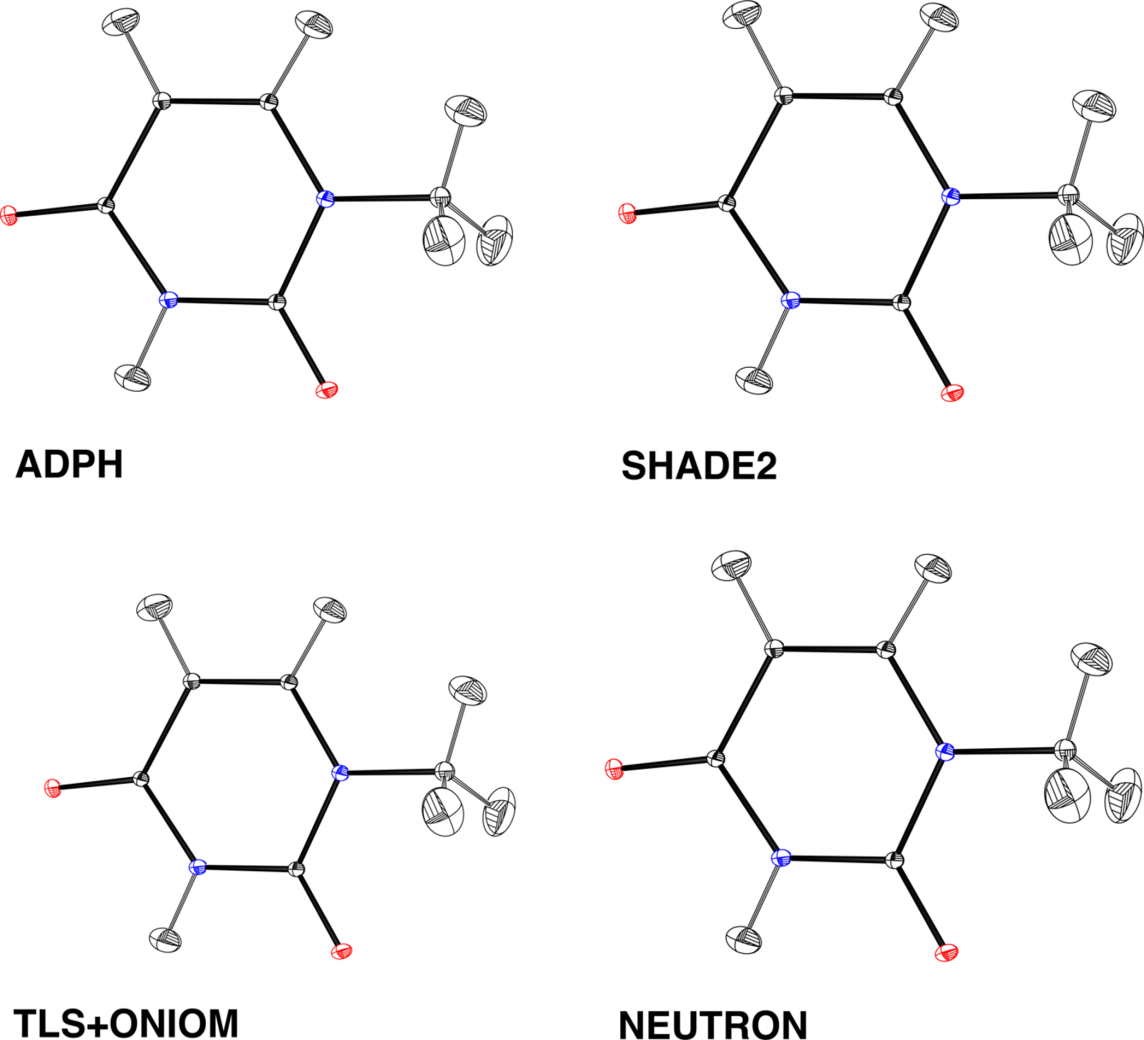
Estimated hydrogen atom ADPs for 1-methyluracil using various approaches.

Several approaches to estimate hydrogen ADPs have been proposed, *e.g.ADPH* (Roversi & Destro, 2004 ▸), *TLS+ONIOM* (Whitten & Spackman, 2006 ▸), *APD* (Lübben *et al.*, 2015 ▸) and *SHADE3* (Madsen & Hoser, 2014 ▸). The *ADPH*, *SHADE* and *TLS+ONIOM* approaches have been compared by Munshi *et al.* (2008 ▸). They differ primarily in the way the internal motion is estimated. The ADPs of hydrogen atoms in 1-methyluracil based on these approaches are compared in Fig. 6 ▸. All approaches were found to be in good agreement with the ADPs based on neutron diffraction experiments. A full periodic DFT approach in the quasi-harmonic approximation can yield very similar results (Mroz *et al.*, 2021 ▸).

## 10. Extracting physical properties from ADP analysis

When the ADPs are accurately determined and there is no sign of disorder they might be used for estimation of thermodynamic properties. This approach can be traced back to Cruickshank (1956*d* ▸), who estimated the entropy of crystalline naphthalene in 1956. In a similar vein, Madsen and Larsen used frequencies obtained from TLS for estimation of the vibrational entropy of a solid (Madsen & Larsen, 2007 ▸). They applied this procedure to obtain the difference in vibrational entropy for the epimeric compounds xylitol and ribitol. Subsequently, this approach was applied for polymorphs of 2-pyridine­carboxaldehyde hydrazones (Mazur *et al.*, 2016 ▸) as well as nicotinamide and pyrazinamide (Jarzembska *et al.*, 2014 ▸).

Meanwhile Bürgi and Capelli developed a new approach, the so-called normal-mode coordinates analysis (NKA) (Capelli *et al.*, 2000 ▸). This approach utilizes multi-temperature single-crystal diffraction measurements to learn about system dynamics from the temperature evolution of ADPs. A common model, which includes both intermolecular and intramolecular motion, is fitted by least squares against ADPs obtained at different temperatures. Anharmonicity is taken into account via a Grüneisen parameter. The NKA approach was applied for naphtalene multi-temperature data and the heat capacity for naphthalene was obtained from the resulting frequencies of normal modes (Capelli *et al.*, 2006 ▸). Aree, Bürgi and their colleagues conducted an extensive study of multi-temperature datasets for three glycine polymorphs (Aree *et al.*, 2012 ▸; Aree *et al.*, 2013 ▸; Aree *et al.*, 2014 ▸), applying synchrotron radiation data to various charge density models to derive optimal atomic displacement parameters and utilizing the NKA model to analyse the thermodynamics of all solid forms. When using the NKA, the investigator must provide an initial model of normal modes and next decide which frequencies to obtain from NKA and which to derive through computational methods. Their findings indicate that achieving more accurate predictions of thermodynamic properties and relative stability in polymorphic systems requires both precise calculations of lattice energy and a refined description of lattice vibrations, including zero-point energy considerations.

## 11. Alternative approach to refining atomic motion using quantum crystallography

The refinement of ADPs assumes that each atom vibrates independently in a harmonic mean field of the surrounding atoms. The frequent use of rigid-bond restraints, analysis of rigid-body motion and use of Gram–Charlier parameters to describe anharmonicity bear witness to the fact that this is a crude assumption. Nevertheless, it is a very useful model that provides a very stable least-squares refinement and precise atomic positions.

Because the thermal contributions to the Debye–Waller factors correspond to the average motion of each atom over the entire crystal, it is not possible to directly extract information on atomic correlation from the elastic diffraction data. Furthermore, it is difficult to come up with convincing models of correlated motion: it will always be a matter of interpretation and the researcher will propose case-by-case models. To circumvent this problem, the model could be based on *ab initio* calculations. In 2016 we proposed a method to refine selected vibrational frequencies of a lattice-dynamical model derived from periodic DFT calculations against single-crystal diffraction data, a technique known as normal-mode refinement (NoMoRe) (Hoser & Madsen, 2016 ▸). This approach was first tested on urea and subsequently applied to l-alanine, naphthalene and xylitol (Hoser & Madsen, 2017 ▸). It was demonstrated that the heat capacity of naphthalene could be obtained in good agreement with calorimetric measurements. Following this, comparable results for heat capacities were obtained for urea, as well as for the alpha and beta polymorphs of glycine, benzoic acid and 4-hy­droxy­benzoic acid after applying NoMoRe (Hoser *et al.*, 2021 ▸). Additionally, NoMoRe was employed to estimate the vibrational contributions to the free energy for various polymorphic systems, including pyrazinamide (Hoser *et al.*, 2022 ▸) and l-pyroglutamic acid (Hoser *et al.*, 2025 ▸). When the ADPs are affected by disorder, the entropy should be evaluated using different equations (Phillips & Walker, 2023 ▸). This observation calls for using a multi-temperature approach to distinguish disorder from thermal vibrations. Such an approach was used in the case of di­methyl 3,6-di­chloro-2,5-di­hydroxy­terephthalate (Kofoed *et al.*, 2019 ▸). While initial studies related to NoMoRe utilized the independent atom model (IAM) for electron density modelling, the approach has since been enhanced, allowing users the flexibility to choose between IAM, HAR or TAAM for the static electron density representation (Butkiewicz *et al.*, 2025 ▸). The NoMoRe approach reduces the number of parameters used to describe the thermal motion significantly and provides an attempt to include the correlated atomic motion in the model, however, at the expense of higher residual densities and larger agreement indices than obtained with the standard ADP refinement (Sovago *et al.*, 2020 ▸). We have no clear answer to the reason for these observations, but it may simply imply that the notion of having one well defined crystal lattice is very far from the situation in real crystals where the microstructure may impose many kinds of local disorder and vibrational behaviour.

## 12. Outlook

The advent of large computational power and mature *ab initio* calculation methods calls for a more integrated approach between quantum calculations and crystallographic analysis. This integration has already been successfully achieved in the description of static charge densities, for instance, through the use of aspherical form factors such as HAR and TAAM.

However, when it comes to nuclear motion – derived from either neutron, X-ray or electron diffraction experiments – we see a need to move beyond the traditional ADPs towards models that more accurately reflect the physical reality in crystals, specifically the correlated atomic motion. We believe that *ab initio* calculations can provide information which is very difficult, or impossible, to extract from standard crystallographic measurements. The NoMoRe approach represents an attempt in this direction, but there is definitely room for further improvement and innovation. For example, there is considerable information in diffuse scattering patterns that often accompany the Bragg scattering that is used in standard crystallography. Thermal diffuse scattering manifests itself as a non-uniform contribution to Bragg peaks, and could be a very interesting phenomenon to consider for complementing the normal-mode refinement with information on the acoustic phonons, in a similar approach to the extraction of information on elastic constants (Wehinger *et al.*, 2017 ▸). Alternative approaches to modelling of atomic motion are also needed in the case of low-resolution data. With the advent of deep-learning techniques to solve the phase problem (Larsen *et al.*, 2024 ▸), structures can be determined at 2 Å resolution. The amount of data at this resolution does not allow refinement of individual ADPs for each atom, and restrained models, or models describing the motion of the full molecule or parts of it, will be needed. The normal-mode refinement is very computing intensive due to the use of periodic DFT calculations. Application of accurate empirical force fields or force fields based on machine-learning algorithms might be an interesting area to explore, either to build a lattice-dynamical model or to investigate the use of molecular dynamics simulations that can better incorporate effects such as anharmonicity and disorder.
